# Novel Predictors of Stroke-Associated Pneumonia: A Single Center Analysis

**DOI:** 10.3389/fneur.2022.857420

**Published:** 2022-03-30

**Authors:** Ya-ming Li, Li Zhao, Yue-guang Liu, Yang Lu, Jing-zhu Yao, Chun-ju Li, Wei Lu, Jian-hua Xu

**Affiliations:** Department of Neurology, Jiading District Central Hospital Affiliated Shanghai University of Medicine & Health Sciences, Shanghai, China

**Keywords:** stroke, infection, pneumonia, international normalized ratio, walking ability

## Abstract

Stroke-associated pneumonia (SAP) is a common cause of disability or death. Although the researches on SAP have been relatively mature, the method that can predict SAP with great accuracy has not yet been determined. It is necessary to discover new predictors to construct a more accurate predictive model for SAP. We continuously collected 2,366 patients with acute ischemic stroke, and then divided them into the SAP group and non-SAP group. Data were recorded at admission. Through univariate analyses and multivariate regression analyses of the data, the new predictive factors and the predictive model of SAP were determined. The receiver operating characteristic (ROC) curve and the corresponding area under the curve (AUC) were used to measure their predictive accuracy. Of the 2,366 patients, 459 were diagnosed with SAP. International normalized ratio (INR) (odds ratio = 37.981; 95% confidence interval, 7.487–192.665; *P* < 0.001), age and dysphagia were independent risk factors of SAP. However, walking ability within 48 h of admission (WA) (odds ratio = 0.395; 95% confidence interval, 0.287–0.543; *P* < 0.001) was a protective factor of SAP. Different predictors and the predictive model all could predict SAP (*P* < 0.001). The predictive power of the model (AUC: 0.851) which included age, homocysteine, INR, history of chronic obstructive pulmonary disease (COPD), dysphagia, and WA was greater than that of age (AUC: 0.738) and INR (AUC: 0.685). Finally, we found that a higher INR and no WA could predict SAP in patients with acute ischemic stroke. In addition, we designed a simple and practical predictive model for SAP, which showed relatively good accuracy. These findings might help identify high-risk patients with SAP and provide a reference for the timely use of preventive antibiotics.

## Introduction

Stroke-associated pneumonia (newly developed pneumonia following stroke onset) is a common complication after stroke, with a reported incidence of 2.4% to 47% (1–3). Stroke-associated pneumonia (SAP) can worsen stroke outcomes, increase the occurrence of severe disabilities, and even cause death (4–8). There have been many studies on predicting SAP before. By summarizing a large number of studies, the predictors of SAP include age, sex, smoking, pre-stroke modified Rankin Scale (mRS), National Institutes of Health Stroke Scale (NIHSS), dysphagia, history of various diseases (e.g., atrial fibrillation, cardiac valve disease, chronic obstructive pulmonary disease, congestive heart failure, diabetes, etc.), and predictors in the blood (e.g., interleukin-6, interleukin-10, procalcitonin, C-reactive protein, leukocyte count, lymphocyte count, neutrophil-to-lymphocyte ratio, fibrinogen, etc.) (9–17). Although the researches on SAP have been relatively mature, the method that can predict SAP with great accuracy has not yet been determined. Moreover, for patients with acute ischemic stroke, routine examinations at admission have much more indicators than these predictive indicators. Therefore, it is necessary to discover new predictors in the routine examinations of stroke patients, and then combine these new factors with known predictors to form an accurate SAP prediction method.

In this study, we attempted to collect undiscovered blood indicators that may be related to SAP in patients with acute ischemic stroke. Then, we analyzed the indicators to determine whether there were new predictors. Furthermore, we tried to use these new predictors, combined with the patient's demographic characteristics, disease history, and stroke-related scores to design a simple and practical method for predicting SAP.

## Methods

### Patients and Population

Patients with acute ischemic stroke admitted to the stroke unit of the department of neurology of Jiading District Central Hospital Affiliated Shanghai University of Medicine & Health Sciences from December 21, 2015, to December 21, 2020, were continuously collected. Patients were enrolled in the research if they (1) had an acute-onset focal neurological deficit combined with neuroimaging evidence of cerebral infarction, (2) were hospitalized within 72 h after the onset of the stroke symptoms, (3) did not have an infection within 2 weeks before admission, and (4) gave informed consent. Patients were excluded from the study if they (1) had other causes of new focal neurological deficits (e.g., transient ischemic attack, etc.), (2) had severe liver dysfunctions, (3) were being treated with antibiotics, (4) were using anticoagulant drugs when they were admitted to the hospital, and (5) had coagulopathy or other serious hematologic diseases.

Ethics approval was obtained from the local ethics committee of Jiading District Central Hospital Affiliated Shanghai University of Medicine & Health Sciences (NO. 2021K03), Shanghai, China. This study had obtained informed consent from all participating patients or their legal representatives.

### Clinical Management and Data

Every stroke patient was sent to the dedicated stroke unit. On admission, a professional neurologist would comprehensively determine whether a patient had pre-stroke infections through the blood test reports (including blood routine examination, C-reactive protein (CRP), etc.) in the emergency department and whether the patient had respiratory infection symptoms (e.g., cough, sputum, shortness of breath, fever, etc.) before the onset of stroke symptoms. For a patient without pre-stroke infections, the professional neurologist was responsible for detailed records of the patient's demographic and clinical data which included age, sex, time of onset of stroke symptoms, smoking status, alcoholism, medication use, disease history (e.g., hypertension, diabetes mellitus, atrial fibrillation or cardiac valve disease, chronic obstructive pulmonary disease, cerebral infarction, hyperlipidemia, intracerebral hemorrhage, myocardial infarction, etc.), and stroke-related assessments. The initial severity of stroke was assessed by the trained neurologist using the national institute of health stroke scale (NIHSS) score (18). Dysphagia was assessed by the water swallow test (WST) (19). When the WST evaluation result was >2, it was considered to be dysphagia. The state of neurological function before the onset of the stroke was evaluated using the modified Rankin Scale (mRS) (20). And, every patient's fasting venous blood was drawn within the first 24 h of admission for routine laboratory measurements, including fasting glucose, glycosylated hemoglobin, serum uric acid, low-density lipoprotein, serum urea, homocysteine (HCY), serum creatinine, international normalized ratio (INR) and so on. Moreover, we assessed each patient's walking ability within 48 h of admission (WA). Having WA was defined as a patient who could walk a distance of more than 3 meters on flat ground independently or with partial assistance (needing guardrail support, assistance from others, using crutches). After admission, every patient was examined and treated by professional neurologists following the treatment guidelines. In addition, within 7 days after the onset of stroke symptoms, the body temperature, respiratory symptoms, physical examination, and auxiliary examination results of all patients were observed every day after admission.

### Outcome Measures

Stroke-associated pneumonia (SAP) was defined as the spectrum of lower respiratory tract infections within the first 7 days after the onset of stroke (21). Furthermore, the spectrum was defined as fever (>38°C) and/or leucopenia (< 4000 ×109/L cells) or leukocytosis (> 12000 ×109/L cells), and at least two of the following: (1) New onset of purulent sputum, change in the character of sputum, or increased respiratory secretions, or increased suctioning requirements; (2) New onset or worsening cough, or dyspnea, or tachypnea; (3) Rales, or bronchial breath sounds; (4) Worsening gas exchange, increased oxygen requirements. In addition, SAP was diagnosed when additionally typical chest X-ray or computed tomography (CT) changes were present (21, 22). The diagnosis of each SAP was confirmed by at least two professional attending physicians using the diagnostic criteria of stroke-associated pneumonia.

### Statistical Analysis

Data were collected and statistically analyzed using SPSS (Statistical Product and Service Solutions) software package version 22.0. According to whether the continuous variables conformed to the normal distributions, the variables were presented as means ± standard deviations or median+interquartile range. Univariate analyses were conducted using Pearson's chi-squared test or Mann-Whitney U test, depending on the character of variables. The determination of independent risk factors and the establishment of the predictive model were achieved through multivariate logistic regression analysis. The likelihood ratio test was used to detect the model. The receiver operating characteristic (ROC) curve and the area under the curve (AUC) were used to measure the predictive ability of different indicators and the SAP model. The Alpha-error level was set at *P* =0.05.

## Results

**Figure 1:**
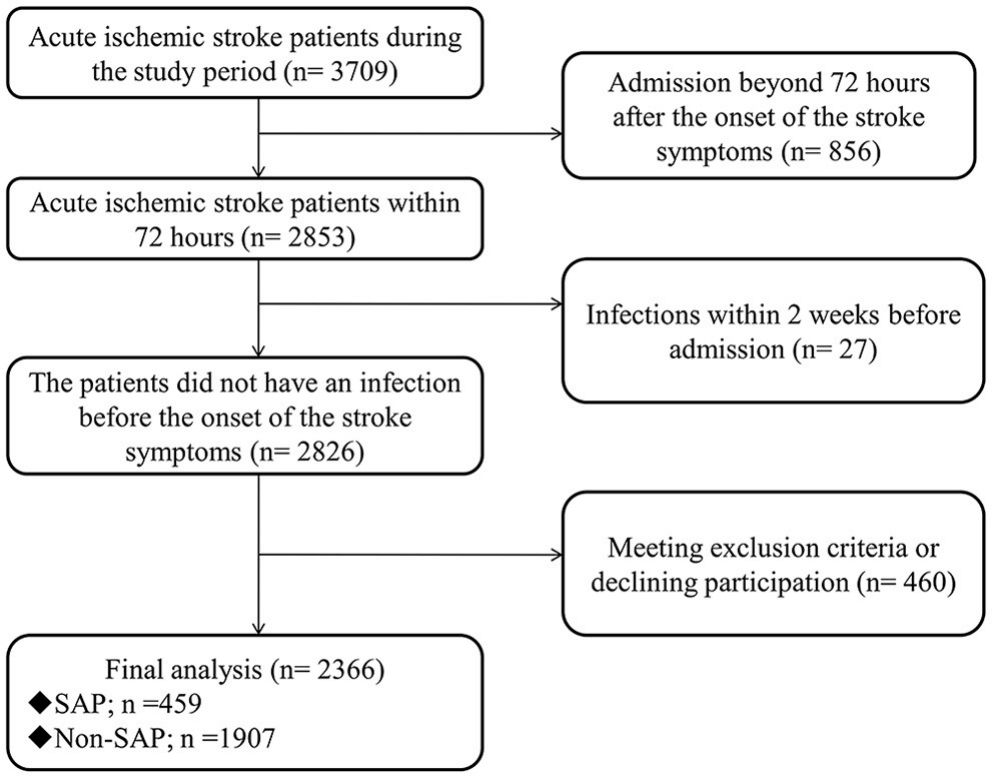
The flow of patients. SAP, stroke-associated pneumonia.

A total of 3,709 patients with acute ischemic stroke were hospitalized during the study period. 2,366 (63.79%) met the inclusion criteria and agreed to participate in this study. These patients included 1,533 males, accounting for 64.8%, and 833 females, accounting for 35.2%. Among the subjects, the oldest was 101 years old, the youngest was 22 years old, and the average and median age are both 71 years old (Figure 1).

The baseline characteristics between groups with and without SAP were presented in Table 1. There were 459 patients who developed SAP within 7 days of stroke, and the incidence rate was 19.40%. Among them, the probability of developing SAP on the first day after the onset of stroke symptoms was the highest (35.7%). Moreover, the lowest (5.7%) was on the seventh day. The average and median time to SAP were 2.82 days and 2 days after the onset of stroke symptoms, respectively. The SAP group presented with older age and higher rates of dysphagia, chronic obstructive pulmonary disease (COPD), cerebral infarction, and atrial fibrillation or cardiac valve disease history. And, factors that accounted for a lower proportion in the SAP group than in the non-SAP group included male, current smoker, alcoholism, and WA. Moreover, the SAP group also had higher admission NIHSS and pre-stroke mRS, and levels of INR, HCY, fasting glucose, serum creatinine, and serum urea than the non-SAP group. In addition, levels of low-density lipoprotein in the SAP group were lower than those in the non-infected group.

**Table 1:** Baseline characteristics of patients with SAP and non-SAP with acute ischemic stroke.

	**Non-SAP (n=1907)**	**SAP (n=459)**	***P* Value**
Sex, male (%)	1273 (66.8)	260 (56.6)	<0.001
Age, y [IQR]	69 [59–78]	82 [73–86]	<0.001
History			
Hypertension (%)	1385 (72.6)	346 (75.4)	0.232
Diabetes mellitus (%)	461 (24.2)	100 (21.8)	0.280
Atrial fibrillation or Cardiac valve disease (%)	124 (6.5)	78 (17.0)	<0.001
COPD (%)	30 (1.6)	18 (3.9)	0.001
Cerebral infarction (%)	364 (19.1)	120 (26.1)	0.001
Alcoholism (%)	366 (19.2)	52 (11.3)	<0.001
Current smoker (%)	559 (29.3)	80 (17.4)	<0.001
Hyperlipidemia (%)	17 (0.9)	6 (1.3)	0.582
Intracerebral hemorrhage (%)	37 (1.9)	14 (3.1)	0.142
Myocardial infarction (%)	16 (0.8)	5 (1.1)	0.813
Assessment at admission			
NIHSS score [IQR]	2.0 [1.0–5.0]	8.0 [3.0–15.0]	<0.001
Dysphagia (%)	95 (5.0)	226 (49.2)	<0.001
WA ^a^ (%)	1345 (70.5)	108 (23.5)	<0.001
Intravenous thrombolysis (%)	350 (18.4)	88 (19.2)	0.685
Pre-stroke mRS score [IQR]	1.0 [1.0–2.0]	2.0 [1.0–2.0]	<0.001
Laboratory indicators			
Homocysteine (umol/L) [IQR]	13.60 [11.00–18.00]	16.50 [12.00–23.10]	<0.001
INR [IQR]	0.92 [0.88–0.97]	0.97 [0.92–1.03]	<0.001
Fasting glucose (m mol/L) [IQR]	5.70 [5.02–7.10]	6.14 [5.30–7.54]	<0.001
Glycosylated hemoglobin (%) [IQR]	5.90 [5.50–6.80]	5.90 [5.50–6.60]	0.613
Low-density lipoprotein (m mol/L) [IQR]	2.79 [2.20–3.41]	2.62 [1.96–3.28]	<0.001
Serum creatinine (umol/L) [IQR]	72.00 [60.50–84.20]	76.80 [64.00–93.00]	<0.001
Serum urea (mmol/L) [IQR]	5.00 [4.20–6.00]	5.70 [4.50–7.40]	<0.001
Serum uric acid (umol/L) [IQR]	315.0 [254.0–382.0]	320.0 [252.0–394.00]	0.297

*IQR, interquartile range; COPD, chronic obstructive pulmonary disease; NIHSS, National Institutes of Health Stroke Scale; WA, walking ability within 48 h of admission; mRS, modified Rankin Scale; INR, international normalized ratio*.

a *Having WA was defined as a patient who could walk a distance of more than 3 meters on flat ground independently or with partial assistance (needing guardrail support, assistance from others, using crutches)*.

Then, we combined the factors with *P*<0.10 in the univariate analyses to perform a multivariate logistic regression analysis. The result showed that age (OR = 1.041; 95% CI, 1.028–1.055; *P*<0.001), INR (OR = 37.981; 95% CI, 7.487–192.665; *P*<0.001), and dysphagia (OR = 7.139; 95% CI, 4.959–10.277; *P*<0.001) were independent risk factors of SAP. However, WA (OR = 0.395; 95% CI, 0.287–0.543; *P*<0.001) was a protective factor of SAP (Table 2).

**Table 2:** Multivariate analysis of factors influencing SAP.

	**B**	***P* Value**	**OR**	**95%CI**
Age	0.041	0.000	1.041	1.028–1.055
Pre-stroke mRS score	0.107	0.170	1.113	0.955–1.298
NIHSS score	0.003	0.835	1.003	0.977–1.029
Fasting glucose	0.037	0.142	1.037	0.988–1.090
Low-density lipoprotein	0.040	0.561	1.040	0.910–1.189
Homocysteine	0.010	0.090	1.010	0.998–1.022
Serum creatinine	0.001	0.800	1.001	0.995–1.006
Serum urea	0.041	0.259	1.041	0.971–1.117
INR	3.637	0.000	37.981	7.487–192.665
COPD	0.678	0.065	1.969	0.960–4.039
Cerebral infarction	−0.036	0.819	0.965	0.711–1.310
Atrial fibrillation or Cardiac valve disease	0.037	0.855	1.038	0.696–1.548
Sex	0.154	0.321	1.166	0.861–1.579
Current smoker	0.076	0.695	1.079	0.738–1.578
Dysphagia	1.966	0.000	7.139	4.959–10.277
WA	−0.929	0.000	0.395	0.287–0.543
Alcoholism	−0.235	0.282	0.791	0.515–1.213
Constant	−8.971	0.000	0.000	

*mRS, modified Rankin Scale; NIHSS, National Institutes of Health Stroke Scale; INR, international normalized ratio; COPD, chronic obstructive pulmonary disease; WA, walking ability within 48 h of admission; B, regression coefficient; OR, odds ratio; CI, confidence interval*.

Next, the indexes of *P* <0.10 in the univariate analysis of the SAP group and the non-SAP group were included in the logistic regression model, and the “Forward: LR” method was used to perform logistic regression. The criteria for entering the model was *P*<0.05, and the criteria for exclusion was *P*>0.10. The variables that eventually entered the model were age, homocysteine, INR, COPD, dysphagia, and WA (Table 3).

**Table 3:** Logistic regression model results.

	**B**	***P* Value**	**OR**
Age	0.041	0.000	1.042
Homocysteine	0.011	0.038	1.011
INR	3.615	0.000	37.136
COPD	0.726	0.046	2.068
Dysphagia	2.052	0.000	7.783
WA	−0.955	0.000	0.385
Constant	−8.077	0.000	0.000

*INR, international normalized ratio; COPD, chronic obstructive pulmonary disease; WA, walking ability within 48 h of admission; B, regression coefficient; OR, odds ratio*.

Subsequently, we assigned the variables that entered the model. The assignment table was shown in Table 4. The regression model was: Logit (P) = −8.077+0.041X_1_+0.011X_2_+3.615X_3_+0.726X_4_+2.052X_5_ −0.955X_6_. It was tested for likelihood ratio, and the test result showed that the regression model was significant (*P* < 0.001). And, the prediction results of the regression model for the occurrence of SAP (a *P*≥0.50 is considered to develop SAP) showed that of the 459 patients with SAP, 217 were correctly predicted; 1,907 were patients without SAP, and 1,823 were correctly predicted. The prediction accuracy of this model is 86.2%.

**Table 4:** Variable assignments.

	**Variable**	**Remarks**
Age	X_1_	–
Homocysteine	X_2_	–
INR	X_3_	–
COPD	X_4_	0 = “NO”, 1 = “Yes”
Dysphagia	X_5_	0 = “NO”, 1 = “Yes”
WA	X_6_	0 = “NO”, 1 = “Yes”

*INR, international normalized ratio; COPD, chronic obstructive pulmonary disease; WA, walking ability within 48 h of admission*.

**Figure 2:**
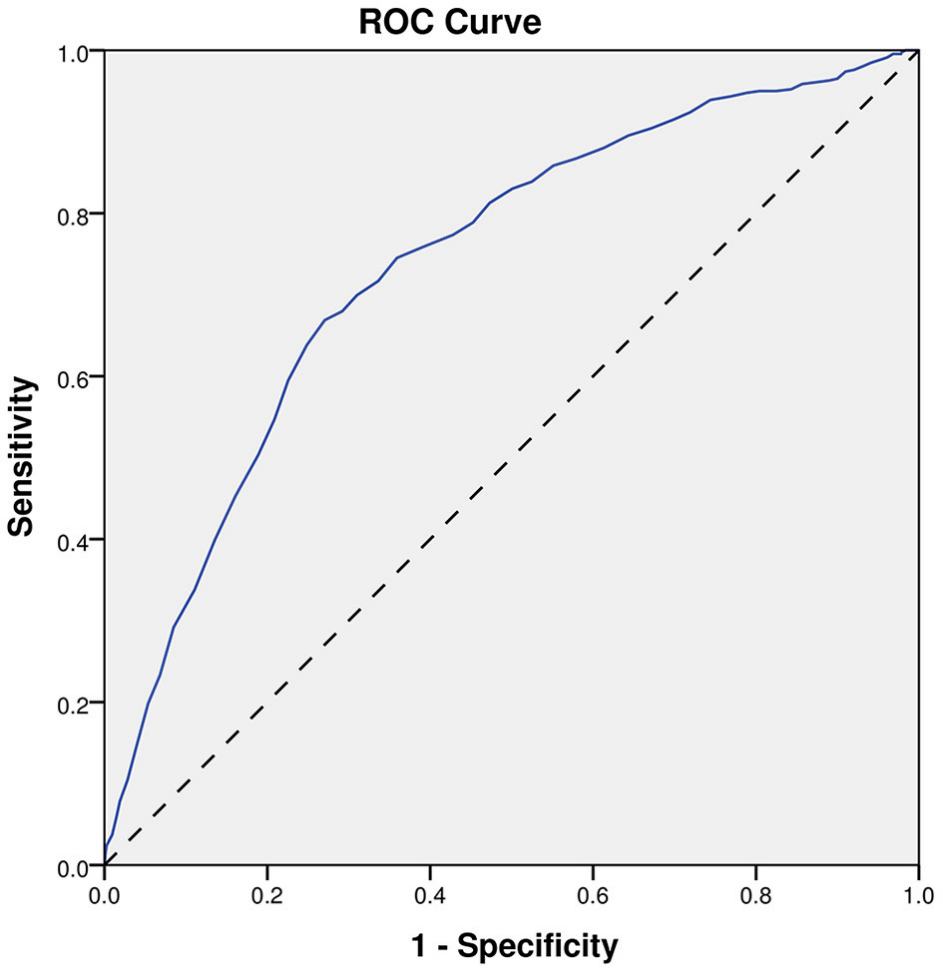
ROC curve of age. ROC, receiver-operating characteristic.

**Figure 3:**
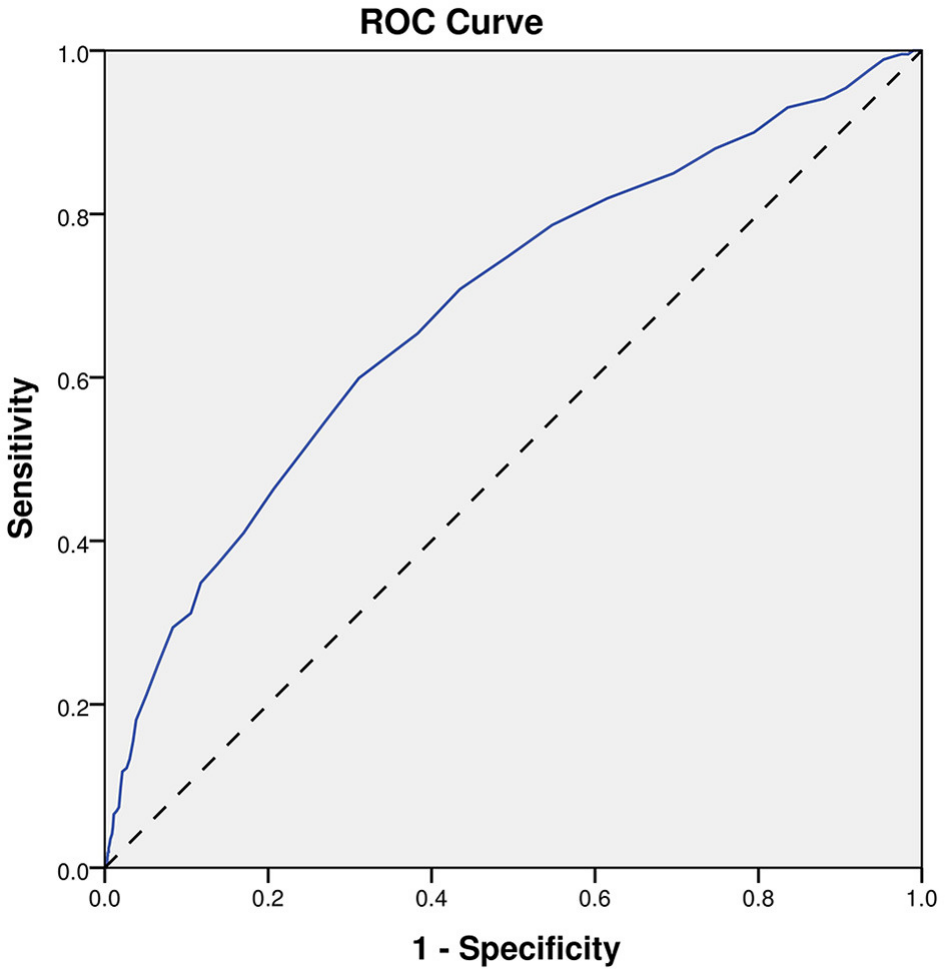
ROC curve of INR. ROC, receiver-operating characteristic.

**Figure 4:**
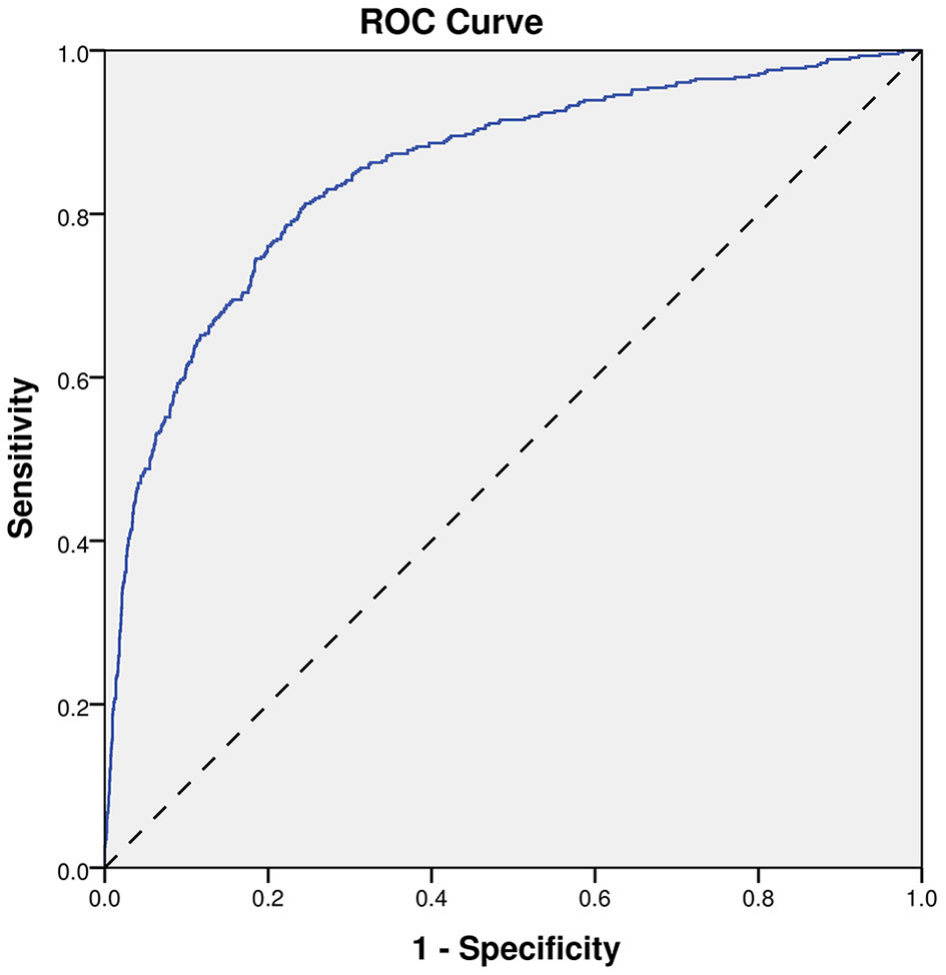
ROC curve of the model. ROC, receiver-operating characteristic.

Finally, the ROC analyses were performed on age, INR, and the model, as shown in Figures 2–4. The results showed that different predictors and the model all could predict SAP (*P* < 0.001). The area under the curve of the model (0.851) is higher than that of age (0.738) and INR (0.685). And, the Youden index for the model was 0.5672. Moreover, the cutoff of the probability (P) was 0.1459. In other words, when *P* = 0.1459, the model had a sensitivity of 81.26% and a specificity of 75.46% for SAP prediction (Table 5).

**Table 5:** Predictive effects of different indicators on SAP.

	**AUC**	**95%CI**	***P* Value**
Age	0.738	0.713–0.763	<0.001
INR	0.685	0.657–0.713	<0.001
Model	0.851	0.831–0.872	<0.001

*INR, international normalized ratio; AUC, area under the curve; CI, confidence interval*.

## Discussion

In this study, we found that a higher INR was associated with SAP in patients with acute ischemic stroke. Furthermore, the high INR at admission may predict the occurrence of SAP.

There are several possible explanations for the finding. One explanation may be that the interaction between the blood coagulation system and pro-inflammatory factors leads to an increase in INR. Firstly, coagulation will be activated after an ischemic stroke. Then, the activation of coagulation leads to the release of pro-inflammatory cytokines and chemokines, which affect inflammation. In addition, Inflammation can also induce the activation of coagulation through pro-inflammatory factors, the most important of which is interleukin-6 (IL-6). IL-6 is a crucial mediator of cytokine-induced coagulation activation (23). Furthermore, early increases of IL-6 concentrations correlated with S100B which was a brain tissue damage marker (24). Also, the increases of IL-6 could induce transcription of the CRP gene and make the CRP level rise rapidly. Then, CRP could bind to the corresponding receptors, resulting in the release of pro-inflammatory cytokines. Next, the interaction between the inflammatory response and the activation of coagulation results in the consumption of coagulation factors, which is manifested as elevated INR (25). Moreover, many previous studies found that pro-inflammatory cytokines (e.g., interleukin-6, CRP, interleukin-10, etc.), S100B, and monocyte chemotactic protein 1 could predict SAP (10, 17, 24). CRP was an acute-phase plasma protein that could be significantly elevated during the acute phase of an inflammatory response (26). Moreover, in the event of bacterial infection, the concentration of CRP in plasma could increase by up to 1000-fold. However, the levels of CRP declined rapidly within 20 h after the end of the stimulation for CRP elevation. In addition, elevated CRP could also produce anti-infective effects through activation of complement and subsequent opsonization of pathogens (27). Therefore, the high INR at admission may be the end-point expression of the release of inflammatory factors. Secondly, a previous study found that the patients with higher INR had a more serious stroke (25) which was related to the increased risk of infection after stroke (11, 28, 29). Moreover, patients with severe stroke were often accompanied by reduced consciousness, which was a risk factor for SAP (30). Therefore, the INR may be used as a marker for patients who are vulnerable to SAP. Last, previous studies found that elevated INR was associated with poor outcomes in patients with acute pulmonary embolism (31), acute ischemic stroke (25), septicemia (32), and acute heart failure (33). And, these diseases may be related to SAP (34). So we consider that elevated INR may be a surrogate sign of a higher burden of risk factors for the development of SAP.

Moreover, our study found that the ability to walk within 48 h of admission might be an independent protective factor for SAP. This result was similar to the result of Chumbler et al. (3). They believed that “found down” was a risk factor for SAP. “Found down” referred to the extent to which, if a patient fell, he/she was unable to get up, and then someone found the patient lying down usually (but not exclusively) on the ground. In our study, the assessment of WA in each patient was performed at the time of physical examination. For a patient who could not cooperate with the WA assessment (such as coma, etc.), we would define that the patient did not have the ability to walk. And, for those few patients who might have WA (such as with severe vertigo, etc.), we would observe the patient's condition changes within 48 of admission and assess the patient's walking ability on time. Also, exercise was good for preventing lung infections (35, 36). Clinical guidelines recommended that patients with acute ischemic stroke who were able to move should take early rehabilitation activities (37). Moreover, among patients with cerebral infarction, the lower the NIHSS score admitted to the hospital, the more likely they were to have WA. In addition, the NIHSS score was statistically different between the SAP group and the non-SAP group, and the same was true for WA. However, in the process of establishing an infection prediction model through the use of binary logistic stepwise regression analyses, the NIHSS score was excluded, but WA was included in the prediction model. Therefore, we believe that WA may have a stronger predictive ability for SAP than the NIHSS score.

Also, we designed a model that might be used to predict SAP. The prediction model included age, HCY, INR, history of COPD, dysphagia, and WA. Among them, age, history of COPD, and dysphagia had been proven by many studies to be used in the prediction of SAP (1, 2, 9). These results were repeated in our trial. In this study, each patient's WST was performed at the time of their physical examination. In addition, we defined dysphagia when a patient was unable to perform WST (e.g., in a coma.etc.). As far as we know, HCY may be used to participate in the SAP prediction for the first time. Previously, Forti P et al. found that hyperhomocysteinemia was associated with a poor outcome in stroke patients (38). Moreover, high HCY could worsen mitochondrial energy metabolism by reducing activities and protein contents of electron transport chain (ETC) components, thereby decreasing adenosine triphosphate (ATP) production (39). ATP was the fundamental energy currency for cellular activity. And, it played an important role in the human body. When pneumonia occurred, ATP would be released into the extracellular space in response to cellular injury and necrosis. Then, it was hydrolyzed into adenosine through a series of enzymes. In an acute setting, adenosine activated its receptors, thereby generating adenosine signals, which could serve an anti-inflammatory and tissue-protective role (40). However, for patients with high HCY, the anti-inflammatory and tissue-protection effects might be worse than those of normal HCY patients. This might be a possible reason that high HCY was a risk factor for SAP. In addition, methicillin-resistant staphylococcus aureus colonized in the nasal cavity was associated with elevated homocysteine levels (41). This might also be a reason why high HCY was related to SAP.

The univariate analyses of this study showed that male, current smoker, and alcoholism might be protective factors for SAP. However, among all patients, 41% and 26.4% of men reported smoking or alcoholism, respectively. In contrast, 1.3% and 1.6% of women, respectively, had a history of smoking or alcoholism. Men were generally thought to be more likely to have a history of smoking and drinking, so these three factors were linked. And, the proportion of men (64.8%) in all patients was higher than that of women (35.2%), which might lead to inaccurate results of the univariate analysis of gender. Therefore, further multivariate analysis was needed to find out the influencing factors of SAP. After multivariate analysis, male, current smoker, and alcoholism were not found to be protective factors for SAP.

This study had some limitations. First of all, although the sample size we included was relatively large, there was still the possibility of selection bias. Therefore, the conclusions might not fully reflect the overall situation. Secondly, the timing of admission might have led to bias. All patients included in our study were admitted to the hospital within 72 h of onset, including some patients admitted to the hospital 24 h after stroke onset. However, all patients had pre-stroke infections ruled out on admission by specialist neurologists based on the patients' symptoms and examination results in the emergency department. And, the proportion of patients who arrived at the emergency department within 24 h of stroke onset was 84.61%. Also, the meantime to the emergency department for all patients was 10.9 h after stroke onset, and the median time was 4.3 h. Thus, the bias was within acceptable ranges. Thirdly, the blood test items included in this study did not include most of the known indicators related to SAP. There might be bias. Fourthly, the time point at which SAP was diagnosed was slightly advanced. Our study found that the highest proportion of patients developed SAP on the first day after stroke. This phenomenon might be related to the diagnostic criteria and diagnosis time of SAP. In our study, the time point at which SAP-related symptoms appeared was regarded as the occurrence time of SAP. There might be a slight bias. Finally, patients using anticoagulant drugs on admission were excluded. This might lead to selection bias. After the study was concluded, we reviewed the hospital records of these patients and found a total of 35 patients eligible for the study. However, only 31 patients had complete data. We then included these 31 patients among 2,366 patients, resulting in new data of 2,397 patients. After that, we performed statistical analyses similar to the original study. And, we found that INR could still predict SAP, and the variables that finally entered the model did not change. Therefore, we believed that excluding patients taking anticoagulants had no significant effects on the results of this study.

## Summary

To the best of our knowledge, our study was the first to find that a high INR might predict SAP events in patients with acute ischemic stroke. In addition, we first proposed walking ability within 48 of admission (WA) as a predictive indicator of SAP and found that this indicator is a protective factor. Furthermore, we designed a simple and practical SAP prediction model which included age, HCY, INR, history of COPD, dysphagia, and WA. And, the model showed good accuracy (simple comparisons of the new model with previous scores were in the Supplementary Material). These findings might help identify high-risk patients with SAP and provide a reference for the timely use of preventive antibiotics.

Subsequent studies should include a larger sample and combine all the reliable predictors found so far to confirm the relationship between the new predictors (INR and WA) and SAP.

## Data Availability Statement

The raw data supporting the conclusions of this article will be made available by the authors, without undue reservation.

## Ethics Statement

Ethics approval was obtained from the local ethics of Jiading District Central Hospital Affiliated Shanghai University of Medicine & Health Sciences (No. 2021K03), Shanghai, China. Written informed consent from the participants was not required to participate in this study in accordance with the national legislation and the institutional requirements.

## Author Contributions

Y-mL wrote the manuscript and sorted out the data. J-hX conceived the study and was the person in charge of this research. LZ and J-hX participated in the revision of the paper. Y-gL conducted the statistical analysis. J-zY, C-jL, and WL were responsible for collecting and proofreading data for this study. YL prepared the report. All authors contributed to the article and approved the submitted version.

## Funding

This study was supported by the National Natural Science Foundation of China (No. 31900700), the Jiading District Medical Key Specialist Construction Plan (No. JDYXZDZK-5), and the Research Project of the Central Hospital of Jiading District (No. ZON201913).

## Conflict of Interest

The authors declare that the research was conducted in the absence of any commercial or financial relationships that could be construed as a potential conflict of interest.

## Publisher's Note

All claims expressed in this article are solely those of the authors and do not necessarily represent those of their affiliated organizations, or those of the publisher, the editors and the reviewers. Any product that may be evaluated in this article, or claim that may be made by its manufacturer, is not guaranteed or endorsed by the publisher.

## Abbreviations

SAP: stroke-associated pneumonia
CRP: C-reactive protein
mRS: modified Rankin Scale
NIHSS: national institute of health stroke scale
WST: water swallow test
HCY: Homocysteine
INR: International normalized ratio
WA: walking ability within 48 h of admission
SPSS: statistical product and service solutions
ROC: receiver-operating characteristic
AUC: area under the curve
COPD: chronic obstructive pulmonary disease
P: probability
B: regression coefficient
OR: odds ratio
CI: confidence interval
ETC: electron transport chain
ATP: adenosine triphosphate.
