# Supplementary material for: Novel Predictors of Stroke-Associated Pneumonia: A Single Center Analysis
Source: Front Neurol. 2022 Mar 30;13:857420. doi: 10.3389/fneur.2022.857420 (PMC9007082; doi:10.3389/fneur.2022.857420)
Supplement: Supplementary file 1 [file Data_Sheet_1.PDF]

Supplementary Material

1 Comparison of the new model with previous scores (A2DS2 Score and ISAN Score)

The factors included in the A2DS2 Score and ISAN Score were both valuable in the univariate analyses of our study. So, we did the multivariate regression analysis and the area under the receiver operating characteristic curves (AUROC) on the factors contained in the A2DS2 Score and ISAN Score respectively and obtained the following conclusions. (Supplementary Figure 1)

1.1 We substituted the 5 items (age, atrial fibrillation, dysphagia, sex, stroke severity) included in the A2DS2 Score into our research data, and the AUROC (95% CI) was 0.838 (0.816-0.859) (P<0.001). However, the AUROC derived from the original study was 0.837 (derivation cohort) and 0.835 (validation cohort) (1).

1.2 We used the same method to substitute the 4 items (pre-stroke independence, sex, age, NIHSS score) included in the ISAN Score into our data to obtain another AUROC (95% CI) of 0.803 (0.780-0.826) (P<0.001). But, the AUROC derived from the original study was 0.79 (derivation cohort) and 0.78 (validation cohort) (2).

Therefore, the area under the curve of our model (0.851) is slightly higher than that of the A2DS2 Score and ISAN Score.

There were three possible reasons for this conclusion. The first was that the factors included in our study not only included demographic, clinical data, and stroke-related assessments, but also some blood indicators. As found in the study by Hotter B et al, some biomarkers could improve prediction slightly (3). The second was that the study population of A2DS2 Score and ISAN Score was German and British who were mostly Caucasian. However, our study population was the yellow race. It might be that ethnic differences accounted for the differences in the prediction accuracy of different methods. The third might be the difference in sample size. Our study only included thousands of patients, while the studies on A2DS2 Score and ISAN Score included more than 10,000 patients.

The A2DS2 Score and ISAN Score contained fewer items than our model and were, therefore, easier to operate. However, our model only included 6 evaluation items, so it was not complicated to operate. Also, the predictors included in all three were relatively easy to obtain, so they all had good feasibility.

2 Supplementary Figure

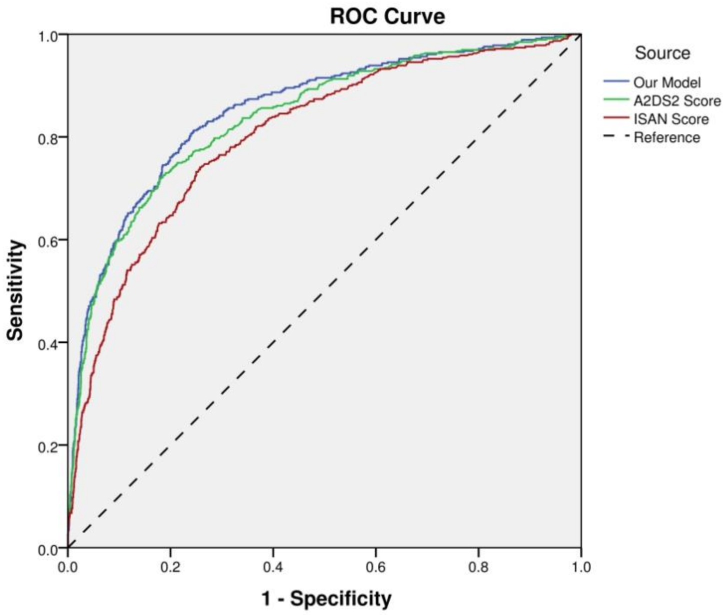

Supplementary Figure 1. ROC curves for discriminatory abilities of the different scores for SAP. ROC, receiver-operating characteristic; SAP, stroke-associated pneumonia.

References

1. Hoffmann S, Malzahn U, Harms H, Koennecke HC, Berger K, Kalic M, et al. Development of a clinical score (A2DS2) to predict pneumonia in acute ischemic stroke. Stroke; a journal of cerebral circulation. 2012;43(10):2617-23.

2. Smith CJ, Bray BD, Hoffman A, Meisel A, Heuschmann PU, Wolfe CD, et al. Can a novel clinical risk score improve pneumonia prediction in acute stroke care? A UK multicenter cohort study. J Am Heart Assoc. 2015;4(1):e001307.

3. Hotter B, Hoffmann S, Ulm L, Meisel C, Bustamante A, Montaner J, et al. External Validation of Five Scores to Predict Stroke-Associated Pneumonia and the Role of Selected Blood Biomarkers. Stroke; a journal of cerebral circulation. 2021;52(1):325-30.
